# Increased PD-1+Tim-3+ exhausted T cells in bone marrow may influence the clinical outcome of patients with AML

**DOI:** 10.1186/s40364-020-0185-8

**Published:** 2020-02-13

**Authors:** Jiaxiong Tan, Zhi Yu, Jingying Huang, Youchun Chen, Shuxin Huang, Danlin Yao, Ling Xu, Yuhong Lu, Shaohua Chen, Yangqiu Li

**Affiliations:** 1 Department of Hematology, First Affiliated Hospital, Jinan University, Guangzhou, 510632 China; 2 Institute of Hematology, School of Medicine, Key Laboratory for Regenerative Medicine of Ministry of Education, Jinan University, Guangzhou, 510632 China

**Keywords:** AML, T cell exhaustion, T cell subset, PD-1, Tim-3

## Abstract

**Background:**

Altered expression of T cell immune inhibitory receptors may result in immunosuppression and associate with the poor prognosis of leukemia patients in which the leukemic bone marrow (BM) microenvironment may contribute to such immunosuppression. We found higher numbers of programmed death-1 (PD-1) + exhausted T cells in peripheral blood (PB) from acute myeloid leukemia (AML) patients. To investigate the leukemic BM influence on immunosuppression, we further compared the distributions of PD-1 and T cell immunoglobulin mucin-3 (Tim-3) and the exhausted T cell phenotype in PB and BM from AML patients and characterized their relationship with clinical outcome.

**Methods:**

PB and BM samples from 15 patients with newly diagnosed AML were collected and analyzed for the expression of PD-1, Tim-3, CD244, and CD57 on CD3+, CD4+, and CD8+ T cells by multicolor flow cytometry.

**Results:**

The proportions of PD-1 + CD3+ and PD-1 + CD8+ T cells were significantly higher in BM compared with PB. Similarly, higher PD-1 *+* CD244 *+* CD3*+* and PD-1 *+* CD244 *+* CD8*+* T cells were found in BM, and an increased tendency for PD-1 + CD244 + CD4+ T cells was also detected in this group. In contrast, increased Tim-3 + CD4+/Tim-3 + CD244 + CD4+ T cells were predominant in BM compared with PB, but there was no statistically significant difference in Tim-3 + CD8+ T cells. Moreover, PD-1 and Tim-3 double-positive CD3+/CD4+/CD8+ T cells were significantly increased in the BM group. In addition, a higher proportion of PD-1 + Tim-3 + CD3+ T cells in the BM and PD-1 + Tim-3 + CD4+ T cells in PB was detected in non-complete remission (NCR) compared with complete remission (CR) patients after first-cycle chemotherapy.

**Conclusions:**

Upregulation of PD-1 and Tim-3 and the exhausted phenotype of CD4+ and CD8+ T cells in the BM of AML patients may contribute to mediating the leukemic immunosuppressive microenvironment, and increased PD-1 + Tim-3+ CD8+ T cells may be related to T cell dysfunction in AML, which may influence clinical outcome.

## Background

Acute myeloid leukemia (AML) is a common type of leukemia in adults arising from hematopoietic stem/progenitor cells with rapid progression and limited treatment management with the exception of the AML-M3 subtype [1, 2]. Although increasing findings have improved understanding of the pathogenesis of AML, standard therapies have not changed for over 30 years. With the recent development of targeted and immunotherapy, a number of ongoing studies aim to produce novel AML therapies, including conventional cytotoxic chemotherapies, genetic and epigenetic targeted therapies, and immunotherapies [3–6]. The T cell immune status of patients is an important factor related to the prognosis of leukemia [7–11]. Significantly, improvement in the clinical outcome of hematological malignancies was demonstrated by using immunotherapies such as CAR-T cell transfusion [12–15]. In addition, checkpoint inhibitor-based monotherapy or combination therapies are being evaluated in clinical trials for leukemia and lymphoma [16, 17]. Although considerable progress has been made in this field, the achievements of ideal effects in solid tumors and acute lymphoblastic leukemia (ALL) have not been realized for AML. Thus, further analysis of the T cell immune dysfunction that exists in AML patients and their causes remain necessary [8]. It is well known that cancer-associated immune suppression can significantly reduce T cell-mediated anticancer immunity. In addition, cancer cells and AML cells are able to escape the host immune surveillance by inactivating cytotoxic T cells (CTLs) [18]. It has been demonstrated that T cell immune inhibitory receptors, also known as immune checkpoint receptors, such as program death-1 (PD-1), T cell immunoglobulin mucin 3 (Tim-3), cytotoxic T lymphocyte-associated molecule-4 (CTLA-4) and T cell lymphocyte activation gene-3 (LAG-3) have increased expression in T cells in patients with newly diagnosed and relapsed AML and murine AML models [19–22]. For example, leukemia progression resulted in increased regulatory T (Treg) cells and PD-1 ligand (PD-L1) + CD8+ T cells at tumor sites in a mouse AML model, and PD-L1 inhibitor treatment could restore the proliferation and cytotoxic function of T cells at tumor sites, reduce AML tumor burden, and increase overall survival [23, 24]. Additionally, Tim-3 was described as an AML stem cell (LSC) antigen, and its ligand galectin-9 (Gal-9) works in an autocrine loop contributing to LSC self-renewal. Moreover, Tim-3 as a trafficker for the secretion of Gal-9 may contribute to AML development [25, 26]. This finding may indicate one of the reasons for the anti-cancer T cell activity impairment in AML. Moreover, increasing co-expression of Tim-3 and PD-1 on CD8+ T cells was reported during AML progression in a murine AML model. IFN-γ, TNF-α, and IL-2 production in PD-1 + Tim-3 + CD8+ T cells was deficient when they responded to PD-L1 in Gal-9 expressing AML cells. PD-1 blockade alone or in combination with blockade of other immune checkpoint proteins can improve the anti-leukemia CD8+ T cell function in murine AML models [2, 18, 24].

The BM immunosuppressive microenvironment in AML can persistently modulate AML cell proliferation and drug resistance by deregulating innate and adaptive immune responses [27]. In our previous studies, we demonstrated alterations in T cell exhaustion with strongly upregulated PD-1 + CD244+ and PD-1 + CD57+ cell populations for both CD4+ and CD8+ T cells in patients with newly diagnosed AML [20]. However, there is limited data on the immunosuppressive effects of the BM microenvironment in AML patients on T cells. Recently, differences in the T cell immune response between BM and PB from AML patients was reported [28]. To further characterize the influence of T cell exhaustion from AML BM, we characterized the different distributions of PD-1, Tim-3, CD244, and CD57 on the T cell subsets in BM and PB from patients with newly diagnosed AML and analyzed associations between the immunosuppression status of different patients and their clinical outcome.

## Materials and methods

### Samples

PB and BM samples were collected from 15 newly diagnosed, untreated AML patients including 4 males and 11 females (median age: 47 years, range: 23–81 years) numbered P1 to P15 (Additional file 2: Table S1), including six PB samples (P2, P4, P5, P7, P13 and P14) that were used in our previous study. In this study, we used the previous samples to combine with BM samples for analysis [20]. The AML subtypes were classified according to the French-American-British (FAB) and World Health Organization (WHO) classification of myeloid neoplasms [29–31]. The patients were followed up after first-cycle chemotherapy and divided in the groups complete remission (CR) and non-complete remission (NCR) according to results from BM smears and flow cytometry analysis. Nine patients (six in CR and three in NCR) could be evaluated.

### Flow cytometry analysis

Cell surface staining analysis for PD-1, Tim-3, CD57, CD244, CD3, CD4, CD8 and CD45 was performed by flow cytometry as previously described [20, 32]. The antibodies used for this study were purchased from Biolegend (San Diego, USA) and BD Biosciences (San Jose, USA).

### Statistical analysis

Statistical analysis of the PB and BM groups was performed using the paired-samples *t* test. The Mann-Whitney two independent sample test was used when analyzing the CR and NCR groups with SPSS software (Version 13.0; SPSS Inc., Chicago, IL, USA). *P*-values < 0.05 were considered statistically significant.

## Results

**Fig. 1:**
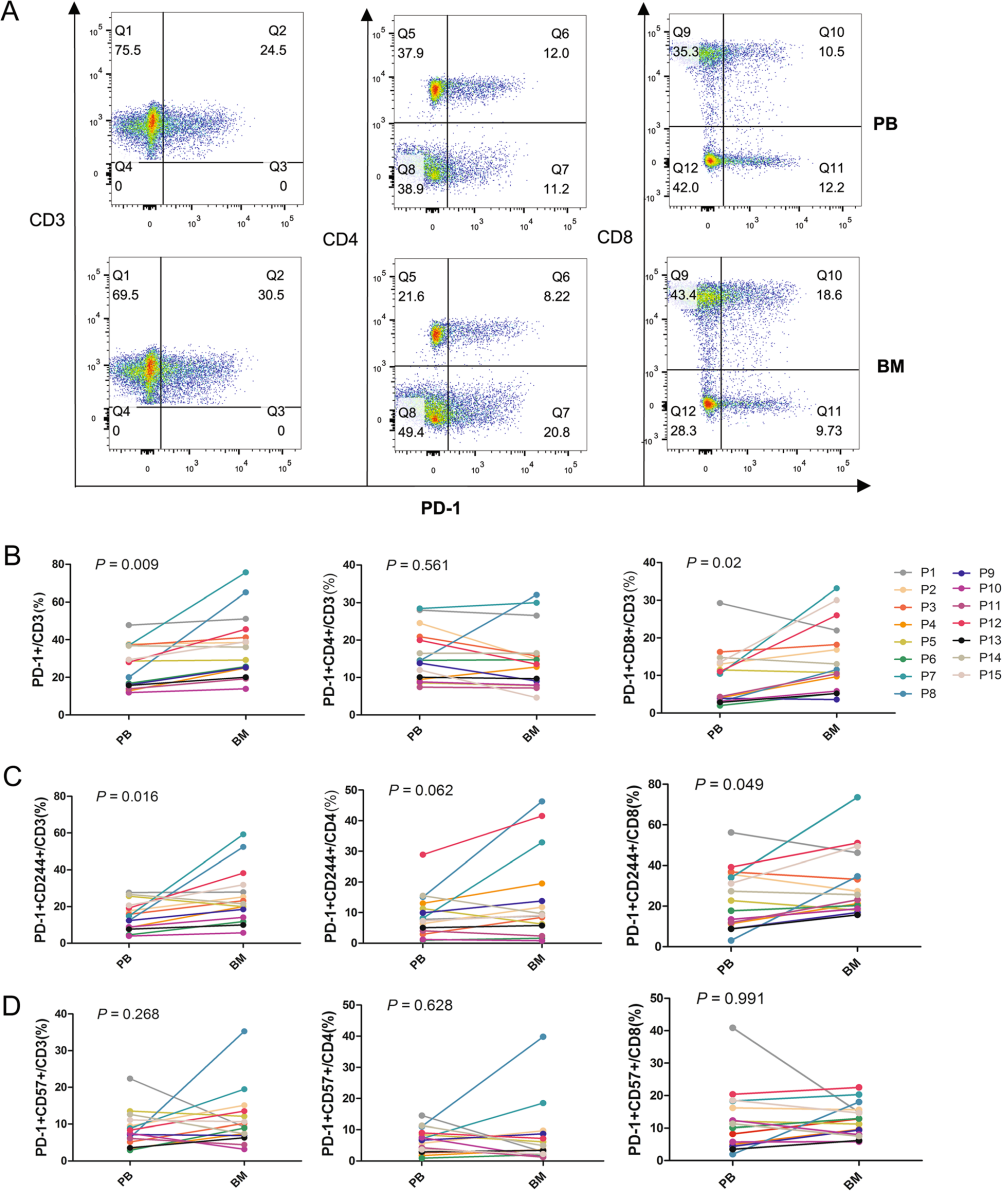
Distribution of PD-1+, CD57+, CD244+ T cells in the CD3+, CD4+ and CD8+ T cell subsets in PB or BM from patients with AML. A: Flow cytometry detection of PD-1 + CD3+, PD-1 + CD4+, and PD-1 + CD8+ T cells in PB and BM in a patient with AML. B: Comparison of the percentage of PD-1 + CD3+, PD-1 + CD4+, PD-1 + CD8+ T cells in PB and BM from 15 patients (P1 to P15) with AML. C: Comparison of the percentage of PD-1 + CD244 + CD3+, PD-1 + CD244 + CD4+, and PD-1 + CD244 + CD8+ T cells in PB and BM from 15 patients with AML. D: Comparison of the percentage of PD-1 + CD57 + CD3+, PD-1 + CD57 + CD4+, and PD1 + CD57 + CD8+ T cells in PB and BM from 15 patients with AML

**Fig. 2:**
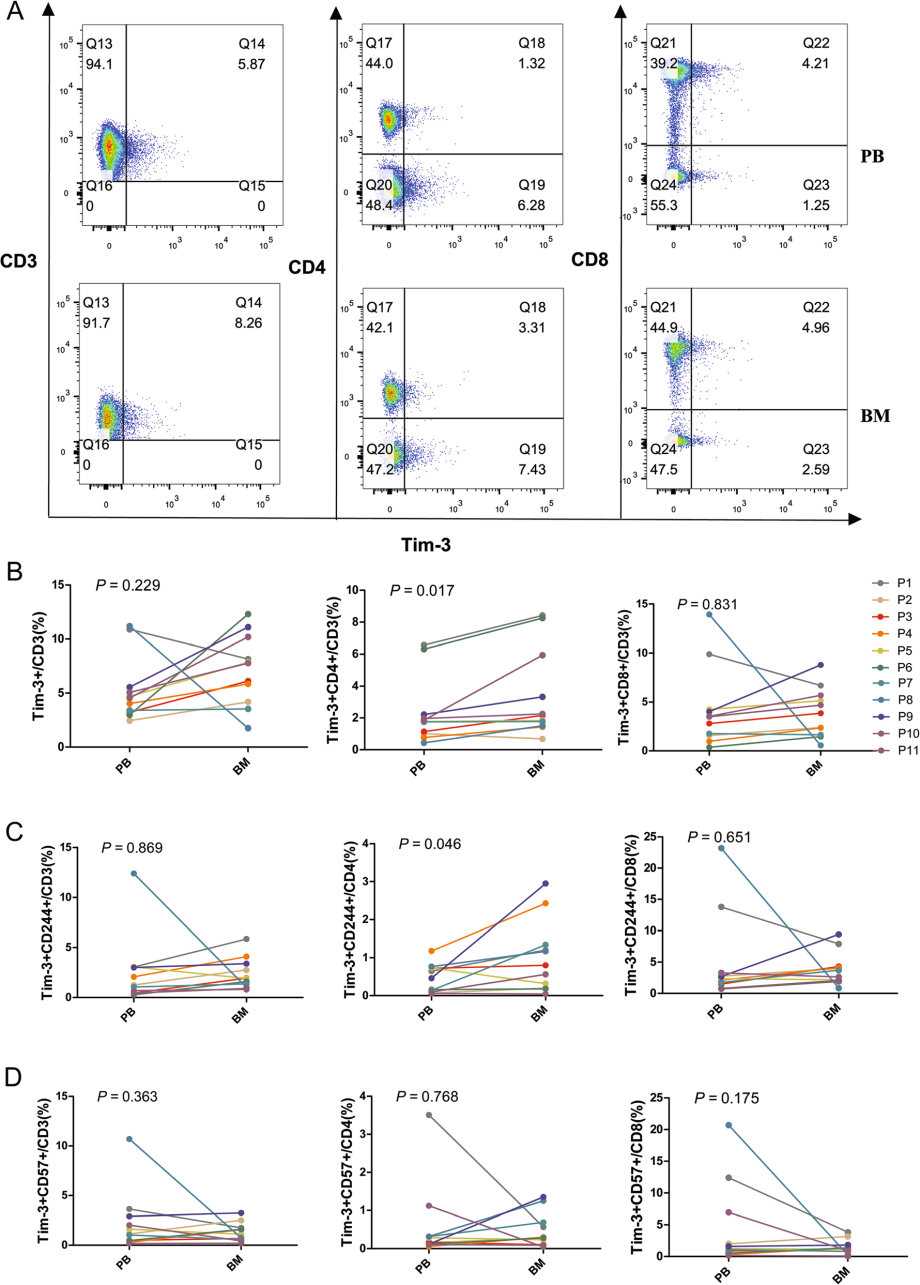
Distribution of Tim-1+, CD57+, CD244+ T cells in CD3+, CD4+ and CD8+ T cell subsets in PB and BM from patients with AML. A: Flow cytometry detection of Tim-3 + CD3+, Tim-3 + CD4+, and Tim-3 + CD8+ T cells in PB and BM from one patient with AML. B: Comparison of the percentage of Tim-3 + CD3+, Tim-3 + CD4+, Tim-3 + CD8+ T cells in PB and BM from 11 patients (P1 to P11) with AML. C: Comparison of the percentage of Tim-3 + CD244 + CD3+, Tim-3 + CD244 + CD4+, and Tim-3 + CD244 + CD8+ T cells in PB and BM from 11 patients with AML. D: Comparison of the percentage of Tim-3 + CD57 + CD3+, Tim-3 + CD57 + CD4+, and Tim-3 + CD57 + CD8+ T cells in PB and BM from 11 patients with AML

In this study, we characterized the distributions of exhausted T cells in PB and BM from AML patients. First, we compared the percentage of CD4+ and CD8+ T cells among the CD3+ T cells in the BM and PB. The results showed a trend toward a high proportion of CD4+ T cells in PB (median: 49.5%) compared with BM (median: 41.9%) (*P =* 0.065), and there was a higher tendency of CD8+ T cells in BM (median: 44.2%) compared with PB (median: 36.9%) (*P =* 0.057) (Additional file 1: Figure S1). Next, we detected the expression of PD-1, Tim-3, CD244, and CD57 in CD3+, CD4+, and CD8+ T cells in the PB and BM from 15 newly diagnosed AML cases (Tim-3+ T cells in 11 cases) (Figs. 1a and 2a). We found significantly increased PD-1 + CD3+ and PD-1 + CD8+ T cells in BM in comparison with PB; however, there was no statistically significant difference in the percentage of PD-1 + CD4+ T cells between the BM and PB (Fig. 1b). We also examined the co-expression of PD-1 or Tim-3 with CD244 and CD57 (exhausted phenotype), and higher percentages of PD-1 + CD244 + CD3+ and PD-1 + CD244 + CD8+ T cells were found in the BM group. Moreover, an increased tendency of PD-1 + CD244 + CD4+ T cells was found in the BM compared with PB (Fig. 1c). However, no significant difference in the percentage of PD-1 + CD57 + CD3+, PD-1 + CD57 + CD4+, and PD-1 + CD57 + CD8+ T cells between BM and PB was found (Fig. 1d). Interestingly, unlike the finding in PD-1+ T cells, Tim-3 + CD4+ and Tim-3 + CD244 + CD4+ T cells were significantly increased in the BM group, but there was no statistically significant difference in the distribution of Tim-3 + CD8+, Tim-3 + CD244 + CD8+, and Tim-3 + CD57 + CD8+ T cells between the BM and PB (Fig. 2). Moreover, it was found that there was a similar alteration trend in the BM in one case who harbored an FLT3-ITD mutation (P9, AML-M4, 43-year-old male) (Additional file 2: Table S1). This patient had a high percentage of Tim-3 + CD3+, Tim-3 + CD244 + CD4+, Tim-3 + CD57 + CD4+, Tim-3 + CD8+, Tim-3 + CD244 + CD8+, and PD-1 + Tim-3 + CD4+ T cells in the BM compared with other AML samples; however, there was no evident difference in PB.

**Fig. 3:**
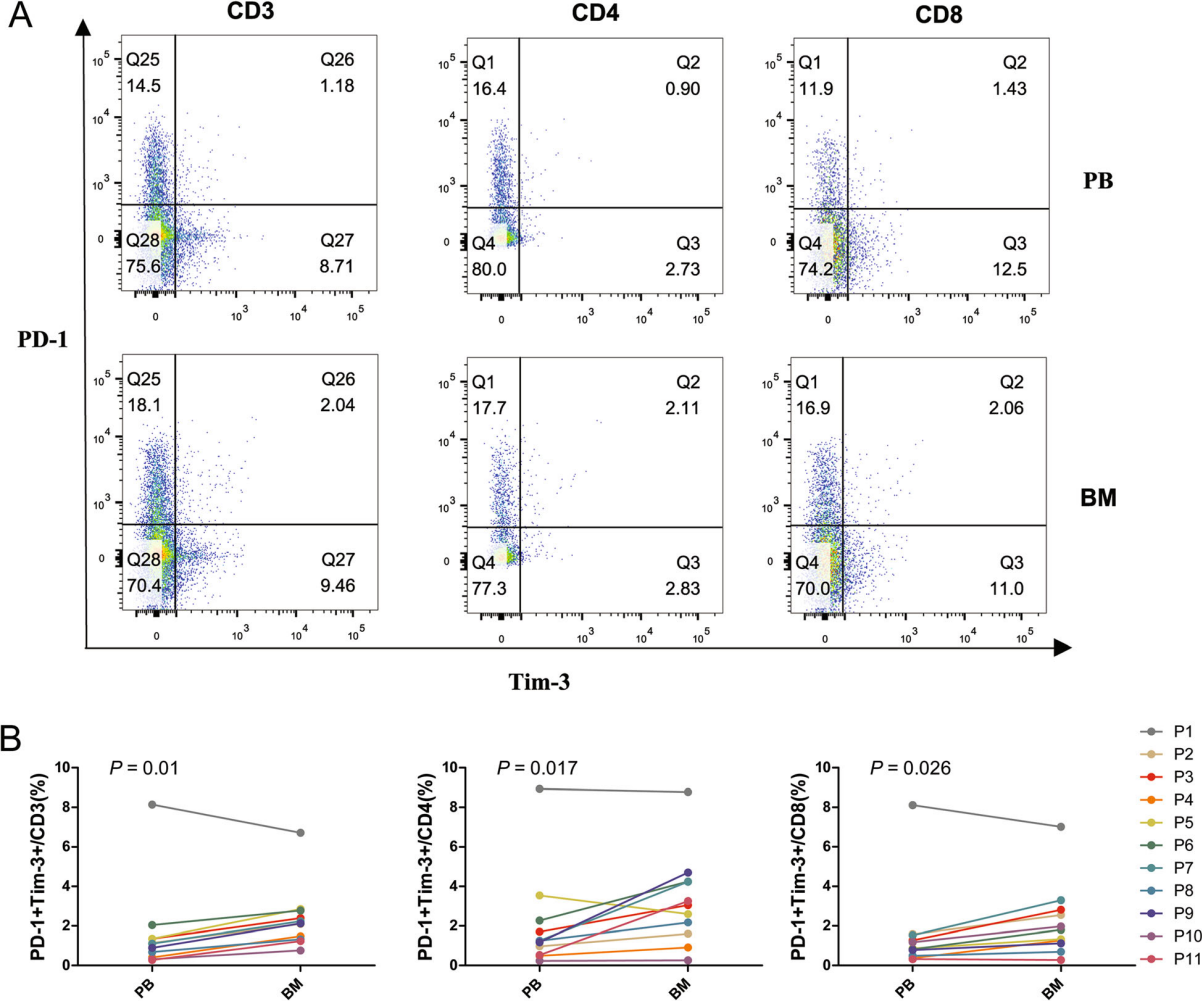
Distribution of PD-1 + Tim-1 + T cells in the CD3+, CD4+, and CD8+ T cell subsets in PB and BM from patients with AML. A: Flow cytometry detection of PD-1 + Tim-3 + CD3+, CD4+ and CD8+ T cells in PB and BM from one patient with AML. B: Comparison of the percentage of PD-1 + Tim-3 + CD3+, CD4+, and CD8+ T cells in PB and BM from 11 patients (P1 to P11) with AML by flow cytometry analysis

When we further analyzed the distribution of PD-1 and Tim-3 double-positive T cells in AML samples, we found that the percentages of PD-1 + Tim-3+ CD3+, CD4+, and CD8+ T cells were significantly higher in BM compared with PB (Fig. 3). The levels of the PD-1 + Tim-3+ T cell percentages were relatively high in both the BM and PB for sample P1 (AML-M2, 61-year-old woman); the broken line in Fig. 3b is distant from the other sample lines.

**Fig. 4:**
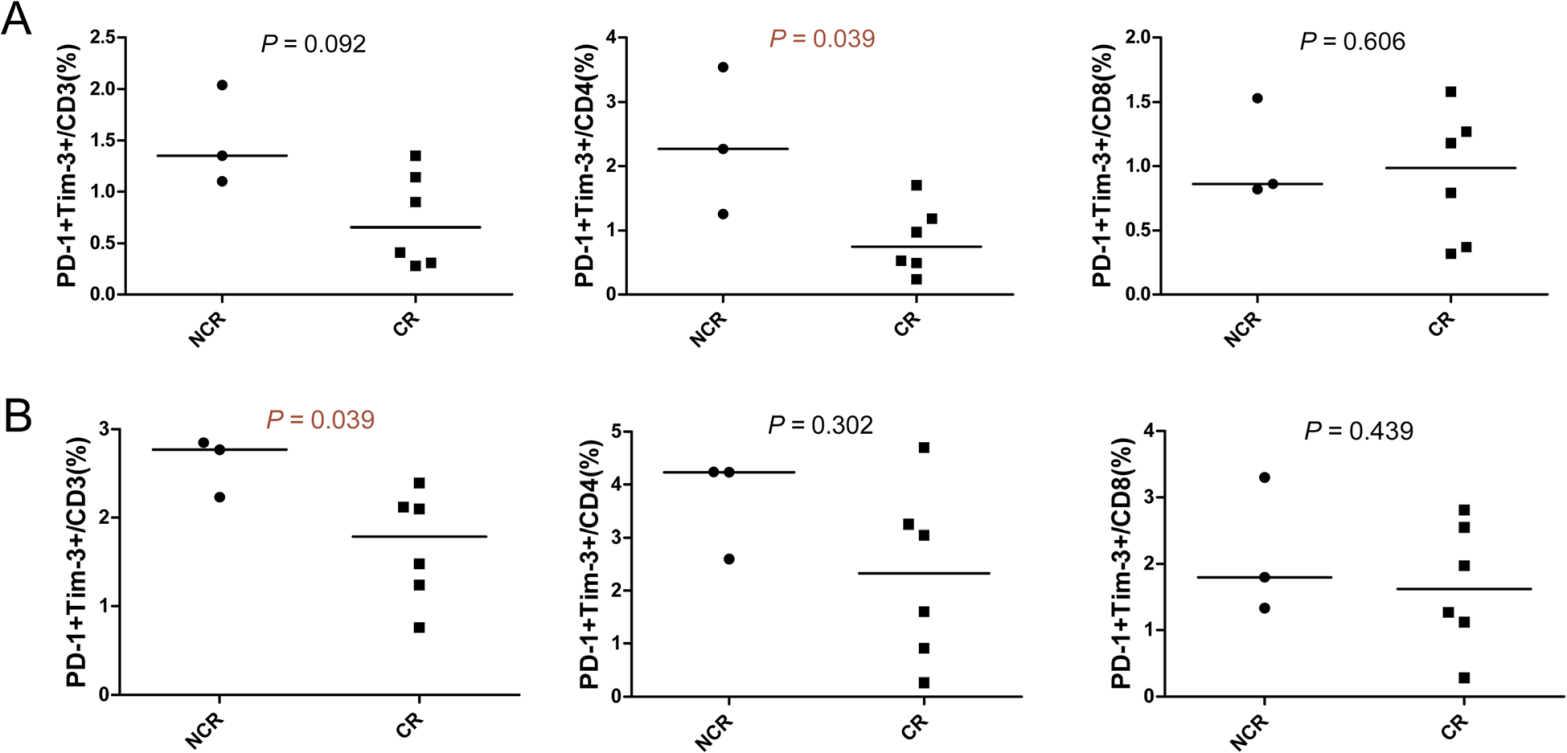
Differences in the distribution of PD-1+ T cells in BM and PB between patients with complete remission and those with non-complete remission after first-cycle chemotherapy as measured by flow cytometry analysis. A: Comparison of the percentage of PD-1 + Tim-3 + CD3+, CD4+, and CD8+ T cells in PB from patients in complete remission (CR, *n* = 6) and non-complete remission (NCR, *n* = 3). B: Comparison of the percentage of PD-1 + Tim-3 + CD3+, CD4+, and CD8+ T cells in BM from patients in the CR and NCR groups

Data from nine patients who underwent chemotherapy in our department after diagnosis (Additional file 2: Table S1) demonstrated a significantly higher percentage of PD-1 + Tim-3 + CD3+ T cells in BM and PD-1 + Tim-3 + CD4+ T cells in PB in the NCR compared with CR group (Fig. 4), and a similar tendency was found in the PB group (the percentage of PD-1 + Tim-3 + CD3+ T cells in the NCR group was 1.350 and 0.655% (median) for the CR group, *P =* 0.092).

## Discussion

Based on previous findings of increased numbers of PD-1+ T cells with the exhaustion phenotype in PB from AML patients [20], we further characterized the influence of the AML BM microenvironment on T cells. We compared differences in the distribution of exhausted T cells between PB and BM from AML patients. First, our findings demonstrate a higher tendency of CD8+ T cells in the BM. In general, the BM contains a higher percentage of CD4+ T cells than PB, and whether this alteration is related to the AML microenvironment or if the CD8+ T cells are activated when migrating to the leukemia BM requires further investigation [33]. Next, the results indicate increased PD-1 + CD3+ and PD-1 + CD8+ T cells in BM from AML patients, which is a similar finding as a recent report on American patients in which a higher percentage of PD-1+ T cells was detected in BM compared with PB in patients with newly diagnosed AML [28]. When we further analyzed co-expression with CD244 and CD57, higher percentages of PD-1 + CD244 + CD3+ and PD-1 + CD244 + CD8+ T cells were also found in the BM group, indicating that PD-1 is frequently co-expressed with the exhausted phenotype on T cells and may be influenced by the AML BM microenvironment, particularly on CD8+ T cells. Moreover, increased exhausted CD8+ T cells results in a reduced anti-leukemia response in patients [28]. Interestingly, Tim-3 + CD4+ and Tim-3 + CD244 + CD4+ T cells primarily accumulated in the BM group, which may suggest that the AML BM microenvironment also has effects on CD4+ T cells, leading to higher expression of Tim-3 with the exhausted phenotype, as it is known that upregulating Tim-3 reduces the activation of T cells [34, 35]. It is interesting that the phenotype of exhausted T cell is distinct in different T cell subsets between BM and PB. Whether a different immunosuppression mechanism may have an influence on the AML BM microenvironment on different T cell subsets remains an open question.

Higher Tim-3 + CD4+ T cells in PB from AML patients significantly correlates with the FLT3-ITD mutation [36]. In this study, only one case had an FLT3-ITD mutation out of 11 AML cases, and we found a similar trend with a high percentage of Tim-3 + CD3+/CD4+/CD8+/CD244+/CD57+ and PD-1 + Tim-3 + CD4+ T cells in the BM, while, unlike the reported results, there was no apparent difference in PB compared with the other AML samples. This finding may be interesting to investigate in a larger cohort.

Increased PD-1 and Tim-3 double-positive T cells in AML BM may provide a global view of the immunosuppression status related to the BM microenvironment. The finding of relatively high PD-1 + Tim-3+ T cells in both the BM and PB in one AML-M2 case is particularly interesting, but the patient did not undergo therapy in our hospital after diagnosis; thus, we were unable to follow their clinical outcome for response after chemotherapy. This patient developed bladder cancer 10 years ago, and whether this is related to the high immunosuppression is worth further investigation of similar cases.

Because the number of Tim-3+ T cells was low, we were unable to analyze the percentages of the PD-1 + Tim-3 + CD244+ and CD57+ T cell subsets. However, combining the results from both the PD-1 + CD244+/CD57+ and Tim-3 + CD244+/CD57+ T cells appeared to demonstrate the same trend. Thus, combination analysis of the expression of both PD-1 and Tim-3 in T cell subsets may better characterize the BM immunosuppression status in AML. Together with our previous finding that memory T cells skew toward terminal differentiation in AML and the leukemic BM niche may have a different impact on central memory T cell (TCM) homing [8], our data demonstrate that the AML BM microenvironment contributes to global T cell dysfunction. To investigate the association between such immunosuppression and clinical outcome, as well as to find any immune biomarker for the prediction of clinical outcome, based on the clinical data, we also compared the different distributions of PD-1 + Tim-3+ T cells in the BM and PB from AML patients with CR and NCR after the first cycle of chemotherapy who we could follow up for clinical outcome. The results indicated that the significantly increased PD-1 + Tim-3 + CD3+ T cells in the BM and PD-1 + Tim-3 + CD4+ T cells in PB was related to NCR, which was in agreement with the finding that PD-1 and Tim-3 co-expression increases during AML progression in an advanced AML mouse model [24]. Thus, it is thought that higher PD-1 + Tim-3 + CD4 + T cells in PB or higher PD-1 + Tim-3 + CD3 + T cells in BM may be one of immune biomarker for poor clinical outcome in AML. These results also indicate that combined PD-1 and Tim-3 blockade may be beneficial in reducing T cell exhaustion and resolving the immunosuppression in AML to overcome chemotherapy resistance. However, the analysis was only based on the limited clinical samples, further investigation is needed to collect and track more samples to confirm the findings.

## Conclusion

Different distributions of PD-1 and Tim-3 together with exhausted CD3+, CD4+, and CD8+ T cells in BM and PB from patients with AML were characterized in this study. Higher expression of PD-1 and Tim-3 in newly diagnosed AML patients was concurrent with exhausted T cells, which may contribute to immune escape in AML and be related to clinical outcome. However, further investigation of the characteristics of exhausted T cells in AML patients, their association with disease relapse, and an evaluation of the effects of checkpoint blockade in different immunosuppression statuses are needed.

## Supplementary information

**Additional file 1: Figure S1.** Differences in the distribution of CD4 + CD3+ and CD8 + CD3+ T cells in BM and PB from 15 patients with AML as measured by flow cytometry analysis.

**Additional file 2: Table S1.** Clinical information for the AML patients used in this study.

## Data Availability

All data generated or analyzed in this study are included in this published article and its supplementary information files.

## Abbreviations

ALL: Acute lymphoblastic leukemia
AML: Acute myeloid leukemia
BM: Bone marrow
CR: Complete remission
CTL: Cytotoxic lymphoid cells
CTLA-4: Cytotoxic T lymphocyte-associated molecule-4
FAB: French-American-British
Gal-9: Galectin-9
HSC: Hematopoietic stem cell
LAG-3: T cell lymphocyte activation gene-3
NCR: Non-complete remission
NK: Natural killer
PB: Peripheral blood
PD-1: Program death-1
PD-L1: Program death ligand-1
RBC: Red blood cell
T_CM_: Central memory T cells
Tim-3: T cell immunoglobulin mucin 3
Treg: Regulatory T cells
WHO: World Health Organization
