# Supplementary figures and images for: Increased PD-1+Tim-3+ exhausted T cells in bone marrow may influence the clinical outcome of patients with AML
Source: Biomark Res. 2020 Feb 13;8:6. doi: 10.1186/s40364-020-0185-8 (PMC7020501; doi:10.1186/s40364-020-0185-8)

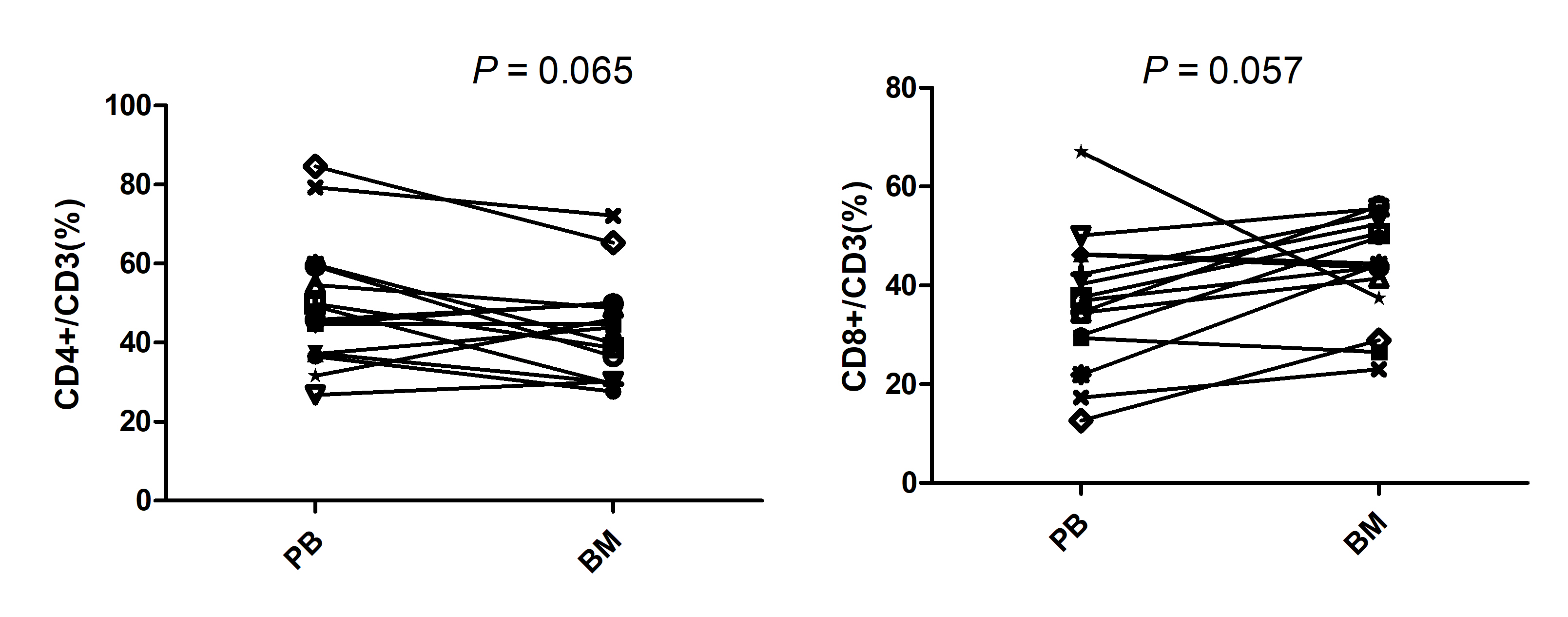

Supplement: Supplementary file 1 — Additional file 1: Figure S1. Differences in the distribution of CD4 + CD3+ and CD8 + CD3+ T cells in BM and PB from 15 patients with AML as measured by flow cytometry analysis. [file 40364_2020_185_MOESM1_ESM.jpg]
