# Supplementary material for: Increased PD-1+Tim-3+ exhausted T cells in bone marrow may influence the clinical outcome of patients with AML
Source: Biomark Res. 2020 Feb 13;8:6. doi: 10.1186/s40364-020-0185-8 (PMC7020501; doi:10.1186/s40364-020-0185-8)
Supplement: Supplementary file 2 — Additional file 2: Table S1. Clinical information for the AML patients used in this study. [file 40364_2020_185_MOESM2_ESM.docx]

**Table 1． Clinical information for the AML patients used in this study**

| Case No. | Sex | Age | Subtype | Diagnosis time | WBC (10^9^/L) | Plt (10^9^/L) | BM blast cell (%) | Genetic alteration | Therapy | Infection/other | Outcome |
| --- | --- | --- | --- | --- | --- | --- | --- | --- | --- | --- | --- |
| P1 | F | 61 | M2 | 10.2016 | 30.34 | 44.1 | 82 | ND | NT | Bladder Cancer (2007) | refusedtherapy |
| P2 | F | 47 | M2 | 10.2016 | 6.9 | 268 | 47 | KIT+, BCORL1 | EA | [extramedullary leukemia](http://www.baidu.com/link?url=U-Ods9EBLPPpxQYfRoRcwoidJOHqOBjQuVtPrgtdjklW8aRhkDaZa7OOAqQOPIIC1NB5CzPP15vScLv-Yf_m5wkwmCGnklms4kqhfcTDy_DB1DXR7HgyBXqnkxJmq2y1&wd=&eqid=c364139b00031203000000065b693861) | CR, survival |
| P3 | M | 26 | M3 | 11.2016 | 1.38 | 24 | 82 | PML-RARA+ | ATRA | Hepatitis B | CR, survival |
| P4 | F | 47 | M3 | 10.2016 | 11.12 | 35 | 76 | PML-RARA+ | ATRA,IDA |  | CR |
| P5 | M | 29 | M3 | 7.3.2017-8.3.2017 | 13.37 | 41.4 | 96 | ND | NT | DIC | Deceased |
| P6 | F | 71 | M2 | 3.2017 | 407.6 | 28 | 96 | ND | NT | Cerebral hemorrhagic shock | Deceased |
| P7 | F | 70 | M5 | 22.11.2016-18.1.2017 | 16.27 | 24 | 75 | TET2, SPSF2, ASXL1 | IA | CNL | NCR,Deceased |
| P8 | F | 81 | M5 | 6.2017 | 17.84 | 21 | 52.5 | ND | NT | Pulmonary infection | refusedtherapy |
| P9 | M | 43 | M4 | 6.2017 | 51.06 | 35.4 | 70.4 | FLT3-ITD+, NPM1 | IA | Pulmonary infection | CR, survival |
| P10 | F | 63 | M4 | 10.2017 | 2.75 | 12 | 37 | TP53 mutation, complex karyotype | Decitabine | [extramedullary leukemia](http://www.baidu.com/link?url=U-Ods9EBLPPpxQYfRoRcwoidJOHqOBjQuVtPrgtdjklW8aRhkDaZa7OOAqQOPIIC1NB5CzPP15vScLv-Yf_m5wkwmCGnklms4kqhfcTDy_DB1DXR7HgyBXqnkxJmq2y1&wd=&eqid=c364139b00031203000000065b693861) | CR, survival |
| P11 | M | 23 | M3 | 10.2017 | 15.1 | 67.2 | 96.5 | PML-RARA | ATRA,IDA |  | CR, survival |
| P12 | F | 59 | M2 | 7.2016 | 4.33 | 4 | 83 | AML1-ETO+, C-Kit/D816, t(8;21)(q22,q21) | EA | Herpes zoster | CR |
| P13 | F | 37 | M3 | 8.2016 | 11.24 | 8 | 94 | FLT3, PML-RARA+ | ATRA，IDA |  | CR |
| P14 | F | 67 | M6 | 2.8.2016-17.11.2017 | 38.5 | 12 | 42 | TET2, ASXL1, P53+, complex karyotype | Decitabine | Pulmonary infection | CR for 8 months, relapse, Deceased |
| P15 | F | 37 | M3 | 9.2016 | 23.18 | 35 | 93.5 | PML-RARA | ATRA, mitoxantrone | Hepatitis B | CR, survival |

Notes: ATRA: all-trans-retinoic acid, BM: bone marrow, CR: complete remission, DIC: disseminated intravascular coagulation, EA: etoposide+ cytarabine, IA: idarubicin+cytosine arabinoside, IDA: Idarubicin, ND: not detected, NT: not treated in our hospital, WBC: white blood cell, Plt: platelet.
